# Metabolic Profiles of Obesity in American Indians: The Strong Heart Family Study

**DOI:** 10.1371/journal.pone.0159548

**Published:** 2016-07-19

**Authors:** Qi Zhao, Yun Zhu, Lyle G. Best, Jason G. Umans, Karan Uppal, ViLinh T. Tran, Dean P. Jones, Elisa T. Lee, Barbara V. Howard, Jinying Zhao

**Affiliations:** 1 Department of Epidemiology, Tulane University School of Public Health and Tropical Medicine, New Orleans, LA, United States of America; 2 Missouri Breaks Industries Research Inc, Timber Lake, SD, United States of America; 3 MedStar Health Research Institute, Hyattsville, MD, United States of America; 4 Division of Pulmonary Medicine, Emory University School of Medicine, Atlanta, GA, United States of America; 5 Center for American Indian Health Research, University of Oklahoma Health Science Center, Oklahoma City, OK, United States of America; 6 Medstar Research Institute and Georgetown and Howard Universities Centers for Translational Sciences, Washington, DC, United States of America; INSERM, FRANCE

## Abstract

Obesity is a typical metabolic disorder resulting from the imbalance between energy intake and expenditure. American Indians suffer disproportionately high rates of obesity and diabetes. The goal of this study is to identify metabolic profiles of obesity in 431 normoglycemic American Indians participating in the Strong Heart Family Study. Using an untargeted liquid chromatography–mass spectrometry, we detected 1,364 distinct *m/z* features matched to known compounds in the current metabolomics databases. We conducted multivariate analysis to identify metabolic profiles for obesity, adjusting for standard obesity indicators. After adjusting for covariates and multiple testing, five metabolites were associated with body mass index and seven were associated with waist circumference. Of them, three were associated with both. Majority of the obesity-related metabolites belongs to lipids, e.g., fatty amides, sphingolipids, prenol lipids, and steroid derivatives. Other identified metabolites are amino acids or peptides. Of the nine identified metabolites, five metabolites (oleoylethanolamide, mannosyl-diinositol-phosphorylceramide, pristanic acid, glutamate, and kynurenine) have been previously implicated in obesity or its related pathways. Future studies are warranted to replicate these findings in larger populations or other ethnic groups.

## Introduction

Overweight and obesity have become global epidemics [1]. Although substantial progress has been made to identify genetic and environmental factors, the mechanisms underlying obesity remain incompletely understood [2]. A comprehensive understanding of its metabolic pathways is critical for developing effective preventive and therapeutic strategies against obesity and its related conditions.

American Indians suffer disproportionately higher rates of obesity and diabetes than other ethnic groups. For instance, the prevalence of obesity was over 40% in American Indians compared to about 27% in non-Hispanic whites [3]. In addition, American Indians are 2 to 3 times more likely to have diabetes than non-Hispanic whites [4]. The prevalence of heart disease among American Indians was also 20% higher than all other U.S. races [4], highlighting the importance of studying this high risk population.

Obesity is typically a metabolic disorder resulting from the imbalance between energy intake and expenditure [5]. Experimental research has demonstrated that altered levels of metabolites in multiple metabolic pathways were associated with obesity, e.g., glucose metabolism [6, 7], lipid metabolism (cholesterol, betaine, acylcarnitines, and carnitine) [8], amino acids (leucine, alanine, ariginine, lysine, and methionine) [8], tricarboxylic acid cycle (pyruvate, citrate, acetoacetate, and acetone) [7], cholines [9], and creatine metabolism (creatine and creatinine) [10]. Altered metabolic profiles, e.g., branched chain amino acids (BCAAs) [11, 12], glutamine, glycine [13], and acylcarnitines [12, 14] have also been associated with obesity and diabetes[15] in human populations. However, most existing studies employed targeted approaches by focusing on a subset of preselected metabolites, but this strategy has limited ability to discover novel disease-related metabolites [11–13]. In addition, previous studies were primarily conducted in European populations. To date, no study has examined the metabolic profile of obesity in American Indians, an ethnically important but traditionally understudied population with high risk of obesity and diabetes [11, 12].

Metabolomics is an emerging high-throughput ‘omics’ technology that can simultaneously quantify a large number of small metabolites in a biological sample. These metabolites serve as substrates or products in metabolic pathways, and are particularly suitable for studying metabolic disorders such as obesity or diabetes. A systematic metabolic profiling using an untargeted metabolomics approach provides a powerful tool to identify novel metabolites and metabolic pathways underlying obesity and related metabolic conditions. In this study, we used an untargeted high-resolution liquid chromatography-mass spectrometry (LC-MS) to identify metabolic profiles for obesity in American Indians participating in the Strong Heart Family Study (SHFS).

## Material and Methods

### Study participants

All study participants were American Indians participating in the SHFS, a family-based prospective study of genetic, metabolic, and behavioral factors for cardiovascular disease (CVD), diabetes, and their risk factors. A detailed description of the study design and methods of the SHFS was published previously [16]. Briefly, a total of 3,665 tribal members (aged 14 years and older) from 94 multiplex families were examined in 2001–2003. All living participants were re-examined about every 5 years and are currently being followed through 2018.

The current study included 431 normoglycemic participants who attended the SHFS clinical examination in 2001–2003. They were randomly selected from a total of 2,117 participants who were free of diabetes and overt CVD at the SHFS clinical examination in 2001–2003. Participants on medications were also excluded from this analysis. Details for the study design and inclusion/exclusion criteria has been described previously [17]. Except for body mass index (BMI) and waist circumference, participants included in the current analysis were not appreciably different from those not included (S1 Table). The SHFS protocol was approved by the Oklahoma Center Indian Health Service institutional review board (IRB), the Dakota Center Indian Health Service IRB, the Arizona Center Indian Health Service IRB, and the MedStar Health Research Institute IRB. It was also approved by the American Indian communities. Informed consent was obtained from each participant or guardians of participants younger than 18 years of age.

### Obesity measurements

Anthropometric measurements including body weight, body height, and waist circumference were conducted with participants wearing light clothing and without shoes using standard methods by trained study staff. BMI was calculated using body weight in kilograms divided by height in meters squared. Waist circumference was measured at the level of the umbilicus while the participant was supine. Participants were classified into three groups according to the WHO definition: normal weight (BMI < 25 kg/m^2^), overweight (25 kg/m^2^ ≤ BMI < 30 kg/m^2^) and obesity (BMI ≥ 30 kg/m^2^). Abdominal obesity was defined as a waist circumference greater than 102 cm in men or greater than 88 cm in women [18].

### Assessment of obesity risk factors

Information on demographics, socioeconomics, and medical history was collected using standard questionnaires. Lifestyle factors including smoking, alcohol intake, physical activity, and habitual diet were examined by personal interview. Smokers were classified as current smokers, former smokers, and nonsmokers. Participants were categorized as current drinkers, former drinkers, and never drinkers by self-reported history of alcohol intake, the type of alcoholic beverages consumed, frequency of alcohol consumption, and average quantity consumed per day and per week. Physical activity was assessed by the mean number of steps per day calculated by wearing a pedometer for 7 consecutive days. Dietary intake was assessed using the block food frequency questionnaire [19]. Fasting plasma glucose, insulin, and lipids were measured by standard methods which were published previously [20]. Insulin resistance was assessed according to the formula: HOMA-IR = fasting glucose (mg/dL) × insulin (μU/mL)/405 [21].

### Metabolic profiling by LC-MS

#### Data acquisition

Relative abundance of fasting plasma metabolites was determined using an untargeted high-resolution LC-MS. Details for laboratory protocols have been previously validated and described elsewhere [22–26]. Briefly, 65 μL plasma sample aliquots were treated with 130 μL acetonitrile (2:1 v/v) containing 3.5 μL of an internal isotopic standard mix [26], placed on ice for 30 min, and centrifuged for 10 min (16,100 x g at 4°C) to remove protein. The supernatant was then removed and placed into autosampler vials. Mass spectral data were acquired using 10 μL of supernatant with a 10 min formic acid/acetonitrile gradient at a flow rate of 0.35 mL/min for the initial 6 min and 0.5 mL/min for the remaining 4 min on a Thermo LTQ-Velos Orbitrap mass spectrometer (Thermo Fisher, San Diego, CA). The first 2-min period consisted of 5% solution A [2% (v/v) formic acid in water], 60% water, and 35% acetonitrile. The final 4-min period was maintained at 5% solution A in acetonitrile. Based on previous research [25], this protocol allows for the measurement of metabolites differing by more than seven orders of magnitude in abundance, and thus we should be able to detect metabolites with a wide range of abundances. The mass spectrometer was set to collect metabolic profile from mass/charge ratio (*m/z)* 85 to 2000 in a positive ionization mode. Three technical replicates were run for each sample using C18 chromatography.

In this study, we used a positive electrospray ionization because previous studies have shown that this mode provides accurate mass matches to metabolites in most pathways included in the Kyoto Encyclopedia of Genes and Genomes (KEGG) human metabolites database [27]. All samples included 14 stable isotopes: [^13^C_6_]-D-glucose, [^15^N]indole, [2-^15^N]-L-lysine dihydrochloride, [^13^C_5_]-L-glutamic acid, [^13^C_7_]-benzoic acid, [3,4-^13^C_2_]cholesterol, [^15^N]-L-tyrosine, [trimethyl-^13^C_3_]caffeine, [^15^N_2_]uracil, [3,3-^13^C_2_]cystine, [1,2-^13^C_2_]palmitic acid, [^15^N, ^13^C_5_]-l-methionine, [^15^N]choline chloride, and 2’-deoxyguanosine-^15^N_2_,^13^C_10_-5’-monophosphate. Quality control was performed based on these internal standards to evaluate mass accuracy in ppm, reproducibility of detection of internal standards and total ion intensity across all samples. To improve data quality, additional filtering steps, as described below, were also applied based on missing values and coefficient of variation (CV).

#### Data pre-processing and quality control

Peak extraction, data alignment, and feature quantification were performed using the adaptive processing software apLCMS, a computer package designed for high-resolution metabolomics data analysis [28]. Data filtering, normalization, and transformation were performed using the computer package MSPrep [29]. Missing data were imputed using the half of the minimum observed value within each metabolite across all samples. Metabolites with extremely high analytical variance, e.g., CV of technical replicates >10%, in our samples were excluded from further analyses. Batch-effect was corrected using the empirical Bayes method ComBat implemented in MSPrep [30]. Potential metabolite identities were determined by performing an online search (10 ppm accuracy) against the Metlin database, the Human Metabolomics Database [31], and the LIPID MAPS structure database [32]. Metabolite annotations were classified into different confidence levels based on the recommended confidence levels assignment for metabolite identification [33].

### Statistical analysis

Prior to statistical analyses, abundance levels of all detected metabolites were log-transformed and standardized to unit variance and zero mean (z-scores). Other continuous covariables were also standardized to z-scores.

To examine the association of metabolites with continuous obesity indices (e.g., BMI or waist circumference), we constructed generalized estimating equation (GEE) models, adjusting for age, sex, study sites, lifestyle factors (smoking, alcohol drinking, and physical activity), and socioeconomic status (education level). The associations of each metabolite with obesity (yes/no) or abdominal obesity (yes/no) were also tested by GEE. The relatedness between family members was accounted for in GEE models. In addition, we further controlled for dietary intake of total daily calories, protein and fat in the GEE models. We also adjusted for insulin resistance in these models because of the strong correlation between obesity and diabetes. Because of the potential high correlations among metabolites, we used the false discovery rate to account for multiple testing, and a *q*-value < 0.05 was considered statistically significant [34].

To test the combined effects of the metabolites showing significant associations with obesity, we constructed a multi-metabolites score using the sum of abundance levels of these metabolites weighted by their regression coefficients obtained from the GEE models. The association between this multi-metabolite score and each obesity measure was tested using GEE, adjusting for covariates listed above. To identify metabolic profiles associated with obesity, we conducted sparse partial least-squares discriminant analysis (sPLS-DA) using the computer package ‘mixOmics’ implemented in R. The sPLS-DA is a supervised, multivariate technique to determine metabolic groups associated with a disease. Compared to sparse discriminant analysis or other wrapper approaches, sPLS-DA is computationally efficient and facilitates interpretability of the results via graphical outputs. The sPLS-DA analysis also allows for adjustments of covariates.

## Results

Table 1 shows the clinical characteristics of study participants attending the SHFS clinical exam in 2001–2003. Compared to participants with normal body weight, those who were overweight or obese had higher levels of triglyceride, total cholesterol, low-density lipoprotein cholesterol, fasting glucose, fasting insulin, and insulin resistance, but lower levels of high-density lipoprotein cholesterol and physical activity. There were no significant differences between the three groups in smoking, drinking, or dietary intake of total daily calories, protein or fat.

**Table 1:** Clinical characteristics of the study participants according to obesity (N = 431).

	Normal	Overweight	Obese	*P* for trend^a^
N	77	97	257	-
Age, years	28.58±13.59	36.99±12.24	34.53±13.25	0.13
Female, %	61.84	59.79	67.32	0.86
Education (high school or higher), %	44.80	68.00	65.70	<0.0001
Body mass index, kg/m^2^	22.17±2.14	27.56±1.36	38.01±6.67	<0.0001
Waist circumference, cm	80.59±7.84	92.96±6.88	115.43±15.28	<0.0001
Current smoker, %	35.53	40.21	34.24	0.44
Current drinker, %	64.47	68.04	67.32	0.69
Physical activity, steps/d	7683±4934	6951±4024	5252±3269	0.01
Dietary protein intake, g/d	96.78±91.35	98.54±86.54	94.36±77.65	0.59
Dietary fat intake, g/d	120.71±100.54	133.09±111.38	122.41±92.79	0.32
Caloric intake, Kcal/d	2843.99±2301.01	2935.79±2342.15	2797.07±1953.04	0.52
Total triglyceride, mg/dL	99.60±37.06	147.72±77.91	154.20±84.25	<0.0001
Total cholesterol, mg/dL	163.86±32.88	187.48±35.80	176.20±31.99	0.009
HDL-cholesterol, mg/dL	58.61±19.22	53.30±15.21	47.64±12.57	<0.0001
LDL-cholesterol, mg/dL	85.41±27.42	105.05±29.71	98.36±27.95	0.05
Fasting glucose, mg/dL	87.58±6.36	90.71±6.94	92.20±7.19	<0.0001
Fasting insulin, uU/mL	8.82±7.51	10.89±5.98	20.28±13.47	<0.0001
HOMA-IR	1.91±1.65	2.46±1.40	4.63±3.11	<0.0001

Abbreviations: HOMA-IR, homeostatic model assessment of insulin resistance.

^a^ Family relatedness was adjusted using GEE models.

A total of 1,364 distinct *m/z* features (CV ≤ 10%) were detected and matched to known compounds in the current metabolomics databases. Five metabolites were significantly associated with BMI and seven were significantly associated with waist circumference. Among these significant metabolites, three were associated with both BMI and waist circumference [oleoylethanolamide (OEA), kynurenine, and mannosyl-diinositol-phosphorylceramide].

Table 2 shows the multivariate associations between significant metabolites and BMI. Of the five metabolites associated with BMI, four metabolites were positively, whereas one was negatively, associated with BMI. In terms of chemical species, one metabolite is amino acids and the other four metabolites are lipids, e.g., fatty amides, sphingolipids, prenol lipids, or steroid derivatives. S2 Table shows the multivariate-adjusted odds ratios (ORs) for obesity status (yes/no) associated with one standard deviation (SD) change in metabolites. The combined effects of these positively associated metabolites on obesity were also statistically significant. Additional adjustments for insulin resistance and dietary intake of fat, protein, and total calories did not appreciably attenuate the observed associations (Table 2 and S2 Table).

**Table 2:** Metabolites associated with body mass index in American Indians.

Matching metabolites	Class	Adduct type	Theoretical m/z	Experimental m/z	Δ ppm^a^	Retention time(s)	Confidence level	Model 1^b^		Model 2^b^	
								β (95% CI)^c^	*P* value	Β (95% CI)^c^	*P* value
***Positively associated metabolites***											
Oleoylethanolamide	Fatty amides	M+H	326.3053	326.3043	3	490	Level 5	0.12 (0.05, 0.18)	4.46×10^−4^	0.13 (0.02, 0.24)	5.77×10^−4^
Kynurenine	Amino acids	M+H	209.0921	209.0909	5	45	Level 1^d^	0.12 (0.05, 0.19)	1.03×10^−4^	0.13 (0.06, 0.20)	1.83×10^−4^
Auxin A	Prenol lipids	M+H	329.2323	329.2319	1	29	Level 5	0.15 (0.07, 0.22)	1.13×10^−4^	0.14 (0.07, 0.21)	2.28×10^−4^
12-Ketoporrigenin	Steroid derivatives	M+Na	469.2924	469.2879	9	496	Level 5	0.18 (0.07, 0.28)	8.47×10^−5^	0.15 (0.07, 0.23)	4.22×10^−4^
*Combined effect*								0.12 (0.07, 0.17)	4.41×10^−6^	0.13 (0.08, 0.18)	1.86×10^−6^
***Negatively associated Metabolites***											
Mannosyl-diinositol-phosphorylceramide	Sphingolipids	M+H	1,358.7725	1358.7724	1	210	Level 5	-0.13 (-0.21,-0.06)	3.01×10^−5^	-0.12 (-0.18,-0.06)	2.28×10^−4^

^a^ Delta in parts per million.

^b^ Model 1 adjusted for age, sex, site, lifestyle (smoking, alcohol drinking, and physical activity), and socioeconomic status (education level); Model 2 further adjusted for dietary caloric, protein, and fat intakes and HOMA-IR.

^c^ Change in BMI per SD change in log-ransformed abundance of metabolites.

^d^ Previously validated with standard and MS/MS.

Table 3 presents the multivariate-adjusted association of metabolites with waist circumference. Of the seven identified metabolites associated with waist circumference, six were positively whereas one was negatively associated with waist circumference. S3 Table shows the ORs for abdominal obesity (yes/no) associated with a 1-SD change in metabolites. Most of the metabolites associated with waist circumference belong to lipids, e.g., fatty amides, prenol lipids, and sphingolipids. Others are amino acids or peptides.

**Table 3:** Metabolites associated with waist circumference in American Indians.

Matching Metabolites	Class	Adduct type	Theoretical m/z	Experimental m/z	Δ ppm^a^	Retention time(s)	Confidence level	Model 1^b^		Model 2^b^	
								β (95% CI)^c^	*P* value	β (95% CI)^c^	*P* value
***Positively associated metabolites***											
Glutamate	Amino acids	M+H	148.0604	148.0594	6	44	Level 1^d^	2.33 (1.08, 3.58)	2.62×10^−4^	2.31 (1.12, 3.49)	2.31×10^−4^
Oleoylethanolamide	Fatty amides	M+H	326.3053	326.3043	3	490	Level 5	1.99 (0.94, 3.03)	1.90×10^−4^	2.09 (1.01, 3.17)	5.32×10^−4^
Kynurenine	Amino acids	M+H	209.0921	209.0909	5	45	Level 1^e^	2.13 (1.02, 3.24)	1.72×10^−4^	2.14 (0.99, 3.29)	2.28×10^−4^
Gly-Val-Arg-Gly	Peptides	M+H	388.2303	388.2304	1	564	Level 5	2.95 (1.40, 4.50)	2.04×10^−4^	2.70 (1.27, 4.14)	1.37×10^−4^
Pristanic acid	Prenol lipids	M+Na	321.2764	321.2761	1	420	Level 5	2.06 (0.95, 3.17)	2.78×10^−4^	2.11 (1.03, 3.19)	2.55×10^−4^
Spirolide E	Prenol lipids	M+Na	748.4759	748.4833	9	426	Level 5	2.33 (1.12, 3.55)	1.62×10^−4^	2.21 (1.01, 3.42)	1.96×10^−4^
*Combined effect*								2.07 (1.62, 2.52)	5.78×10^−6^	2.14 (1.67, 2.60)	5.23×10^−6^
***Negatively associated metabolites***											
Mannosyl-diinositol-phosphorylceramide	Sphingolipids	M+H	1,358.7725	1358.7724	1	210	Level 5	-2.33 (-3.52,-1.15)	1.22×10^−4^	-2.08 (-3.14,-1.02)	2.25×10^−4^

^a^ Delta in parts per million.

^b^ Model 1 adjusted for age, sex, site, lifestyle (smoking, alcohol drinking, and physical activity), and socioeconomic status (education level); Model 2 further adjusted for dietary caloric, protein, and fat intakes and HOMA-IR.

^c^ Change in BMI per SD change in log-ransformed abundance of metabolites.

^d^ The m/z and RT matching with the internal standard.

^e^ Previously validated with standard and MS/MS.

**Fig 1:**
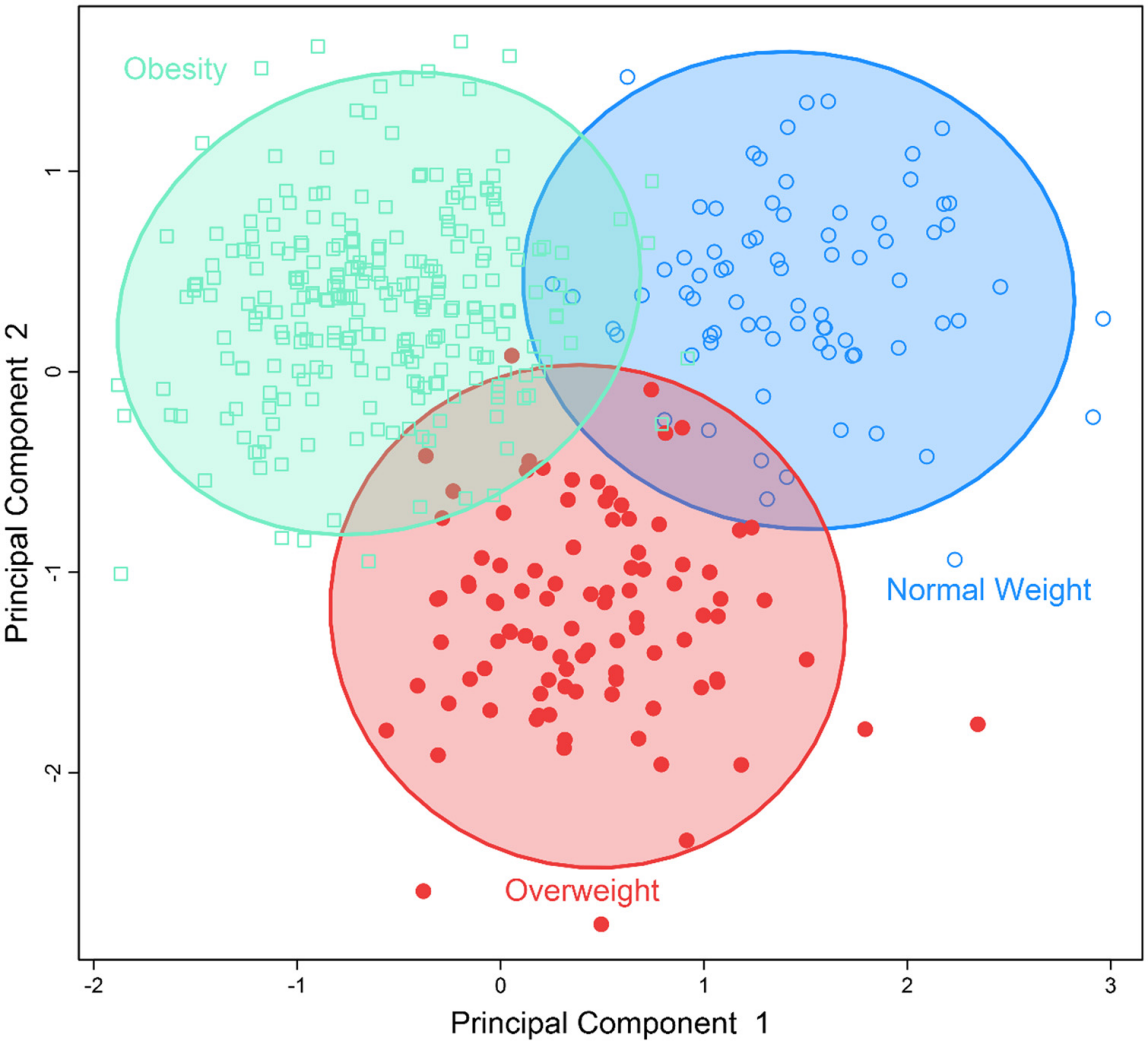
Separation of individuals with normal bodyweight, overweight or obesity using a multi-metabolites score comprising of all metabolites significantly associated with BMI using sparse partial least-squares discriminant analysis.

**Fig 2:**
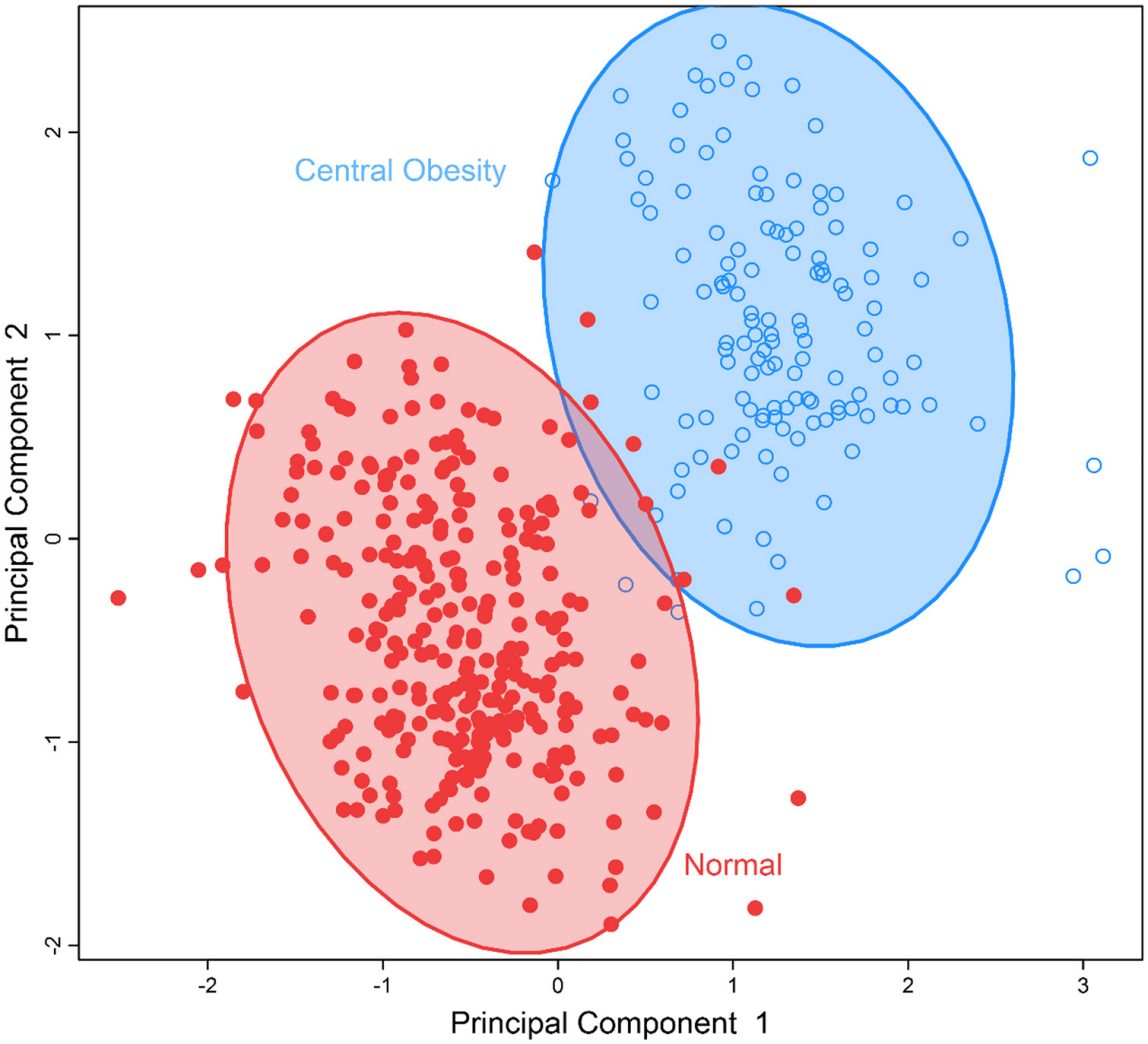
Separation of individuals with normal body weight versus those with abdominal obesity by a multi-metabolites score comprising of all metabolites significantly associated with waist circumference using sparse partial least-squares discriminant analysis.

To identify obesity-related metabolic patterns and to examine the discriminant ability of the identified metabolites in differentiating obese *vs*. nonobese individuals, we conducted multivariate analysis by sPLS-DA. Fig 1 demonstrates that participants who were obese, overweight, or normal body weight could be classified into three separate groups using the five metabolites significantly associated with BMI. Fig 2 demonstrates that the seven metabolites associated with waist circumference could also separate participants into two groups: abdominal obesity vs. non-obese. These results revealed the different metabolic profiles of obese vs. nonobese individuals, and suggest that the identified metabolites could be used to differentiate obese vs. nonobese participants.

## Discussion

Using an untargeted high-resolution LC-MS, we demonstrated that obese vs. nonobese American Indian participants have clearly different metabolic profiles. Specifically, we identified nine plasma metabolites significantly associated with BMI or waist circumference or both. Of these, six metabolites belong to the super class of lipids, including fatty acid amides, prenol lipids, sphingolipids, and steroid derives, and the remaining three are amino acids or peptides. Some of the metabolites, such as OEA, pristanic acid, mannosyl-diinositol-phosphorylceramide, and glutamate, have been previously implicated in obesity-related metabolic pathways [35–39]. Other identified metabolites could be involved in as yet unknown metabolic pathways related to obesity. Of note, the identified associations between metabolites and obesity were independent of known obesity indicators including age, lifestyle, dietary energy intake and insulin resistance. To the best of our knowledge, this is the first study to investigate the metabolic profiles of obesity in American Indians.

In line with previous studies, we found that an elevated level of plasma OEA was positively associated with obesity (both BMI and waist circumference). First, OEA is a biologically active lipid amide synthesized by small intestinal enterocytes during absorption of dietary fat [37]. The release of OEA reduces food intake and induces satiety through activating peroxisome proliferator-activated receptor-α (PPAR-α), which is a transcription factor that belongs to the superfamily of nuclear hormone receptors [40]. PPAR-α is highly expressed in brown adipose tissue and the liver. It functions as a lipid sensor in the liver and recognizes and responses to the influx of fatty acids by stimulating the transcription of numerous genes related to lipid metabolism in the liver, including genes involved in mitochondrial β-oxidation, fatty acid uptake and binding, and lipoprotein assembly and transport [41]. The activation of PPAR-α increases hepatic fatty acid oxidation and decreases the levels of circulating triglycerides that are responsible for adipose cell hypertrophy and hyperplasia, and thus reduces body weight [42]. Second, the identified association between OEA and obesity in our study is in agreement with evidence from experimental studies. For example, in one study, administration of OEA inhibited weight gain in rats [36, 43]. Another study showed that PPARα-deficient mice developed abdominal obesity [44]. Moreover, OEA levels in multiple tissues (e.g., liver, pancreas, adipose tissue) were found to be higher in obese rats compared to normal ones [45]. Third, a recent study in human has also shown that plasma OEA was positively correlated with BMI in obese individuals (BMI ≥ 30 kg/m^2^), although no significant correlation was identified in nonobese individuals [37]. While our finding for the association of OEA with obesity corroborates previous evidence and further highlights the potential importance of OEA in body weight regulation or obesity, the precise mechanism underlying the relationship between OEA and obesity remains to be determined.

In this study, we also found a positive association of pristanic acid with abdominal obesity in American Indians. Pristanic acid is a branched chain fatty acid that an individual can obtain through the consumption of dairy products, ruminant animal fats, and certain fish [46]. Pristanic acid is one of the natural ligands of PPARα [39], and represents the final product of alpha-oxidation of phytanic acid that accumulates in a variety of metabolic disorders [47, 48]. In our study, an elevated level of plasma pristanic acid was significantly associated with abdominal obesity. As pristanic acid could serve as an efficient agonist of PPARα, it is possible that an increased level of pristanic acid could result in decreased activity of PPARα [38], which in turn causes dysregulation of lipid metabolism and body weight [42, 49], thereby contributing to obesity pathogenesis. Further research is warranted to confirm or refute this hypothesis.

Besides OEA and pristanic acid as discussed above, we also found an association of sphingolipids (mannosyl-diinositol-phosphorylceramide) with obesity. Sphingolipids are constituents of cellular membranes that have been involved in cellular signaling processes, vesicle trafficking, and membrane integrity [50, 51]. Previous metabolomic studies have reported that a high level of serum sphingomyelin was associated with obesity in human [52]. Moreover, several plasma sphingolipid chemicals were found to be predictive of cardiovascular and total mortality [53], suggesting a potential role of these lipids in obesity and related conditions.

Amino acids have been previously implicated in obesity [11, 12] and diabetes [12, 15]. In this study, we also detected associations of several amino acids with obesity indices. For instance, an elevated level of plasma glutamate was positively associated with waist circumference in our study population. This finding is in agreement with a previous study showing that glutamate can distinguish lean from obese individuals [12]. Glutamate is an excitatory neurotransmitter in the mammalian central nervous system, and plays an important role in both physiological and pathological processes [35]. Animal studies have shown that monosodium glutamate intake increases the risk of obesity [54], probably through increasing the palatability of food by disrupting the hypothalamic signaling cascade of leptin action [15, 55].

In line with previous research, we also found that kynurenine was significantly associated with both BMI and waist circumference in our study population [56, 57]. Kynurenine is a degradation product of the amino acid tryptophan. The kynurenine pathway is the main route of tryptophan degradation.[58] The enzyme indoleamine 2,3-dioxygenase (IDO) is the major enzyme in the kynurenine pathway that degrades tryptophan to kynurenine. IDO is expressed in many tissues including the adipose tissue and could be induced by pro-inflammatory cytokines, such as TNF-α, IL-1, and IFN-γ.[58] It is known that various pro-inflammatory cytokines could be synthesized and released in human adipose tissue.[59] IDO gene expression is increased in the adipose tissue of individuals with obesity.[60] Serum kynurenine/tryptophan ratio reflects the activity of IDO and this ratio is increased in obesity.[60] Increased IDO activity, essentially caused by chronic immune-mediated inflammation, has been suggested as a key component in the initiation and propagation of obesity. One of the possible mechanisms is that reduced tryptophan mediated by IDO may reduce serotonin production and cause mood disturbances, depression, and impaired satiety ultimately leading to increased caloric uptake and obesity [61].

Previous human studies have reported associations of branched chain amino acids (BCAAs, leucine/isoleucine and valine) with obesity [62], insulin resistance [12, 63], and diabetes [15]. Our study, however, did not detect a significant association of BCAAs with obesity indices. This discrepancy could be attributed to the differences in genetic background and/or lifestyle factors between American Indians and other ethnic groups included in previous studies, because these factors could potentially lead to population-specific metabolic signatures. The lack of replication could also result from the inappropriate exclusion of a large number of metabolites (false negatives) due to multiple testing corrections. Future large-scale metabolomics studies should address this discrepancy.

In addition to the above discussed metabolites, we also found associations of certain prenol lipids (e.g., auxin A and spirolide E), steroid derivatives (e.g., 12-ketoporrigenin), and peptides (e.g., Gly-Val-Arg-Gly) with obesity measures. Biological functions of these metabolites are still unknown. Although these associations need further replication, our findings may unravel novel metabolic pathways implicated in obesity pathogenesis.

BMI is a measure of general obesity, whereas waist circumference reflects abdominal obesity. Waist circumference reflects intra-abdominal fat accumulation, a predictor of adverse metabolic or cardiovascular outcomes independent of BMI [64]. In this study, we identified that several metabolites associated with both types of obesity, but differential metabolomic profiles of general obesity *vs*. abdominal obesity were also identified. These findings may suggest distinct but overlapping pathophysiological mechanisms between general obesity and abdominal obesity. Our results corroborate the differential effects of general *vs*. abdominal obesity on health outcomes [65].

Our study has several limitations. First, metabolites identified in our study are matched to molecular entities within the current metabolomics databases. The precise structures of these newly detected metabolites need to be determined in future studies. Second, although highly correlated, relative abundances instead of absolute concentrations were used as a surrogate for plasma metabolite levels. Third, although we controlled many known risk factors including dietary factors, residual confounding cannot be entirely excluded. Fourth, given the strong correlation between obesity and diabetes, it is highly likely that the identified obesity-associated metabolites might be also related to insulin resistance, a common mechanism underlying both obesity and diabetes. In this study, we additionally adjusted for insulin resistance in the model. It shows the association of the identified metabolites with obesity slightly attenuated but remained statistically significant. Further studies are necessary to clarify the roles of these metabolites in the pathogenesis of obesity and even other diseases. Finally, our results are very preliminary and should not be overinterpreted. In addition, the directions of causality between metabolites and obesity could not be determined in the cross-sectional analysis of the current study. Therefore, replication in larger populations and/or other ethnic groups and even longitudinal studies are warranted. The identified metabolites need to be confirmed by more advanced downstream analyses. Nonetheless, this is the first study to report metabolic profiles of obesity in American Indians. The untargeted high-resolution metabolomics approach enabled a comprehensive analysis of metabolic markers for obesity. The identification of chemicals with known functions involved in body weight regulation, such as OEA, enhances the confidence that some of our findings may represent true metabolites associated with obesity. Moreover, because some obesity-related metabolites identified in our study were also reported to be associated with obesity in other ethnic groups, it seems plausible to hypothesize that our findings could be generalized, at least partially, to other ethnicities.

In summary, this is the first study to identify novel metabolites and metabolic profiles of obesity in American Indians. Our findings highlight the importance of disturbed metabolic pathways, especially dysregulation of lipids, in body weight regulation or obesity pathogenesis. Replication in other populations and functional studies are required to confirm these findings.

## Supporting Information

S1 Fig Mass chromatogram for obesity-related metabolites. (PPTX)

S1 Table Clinical characteristics of SHFS participants who were included compared to those not included in this study. (DOCX)

S2 Table Association between obesity-related metabolites and general obesity in American Indians. (DOCX)

S3 Table Association between abdominal obesity-related metabolites and abdominal obesity in American Indians. (DOCX)
