# Supplementary material for: Metabolic Profiles of Obesity in American Indians: The Strong Heart Family Study
Source: PLoS One. 2016 Jul 19;11(7):e0159548. doi: 10.1371/journal.pone.0159548 (PMC4951134; doi:10.1371/journal.pone.0159548)
Supplement: S1 Fig — (PPTX) [file pone.0159548.s001.pptx]

## Slide 1
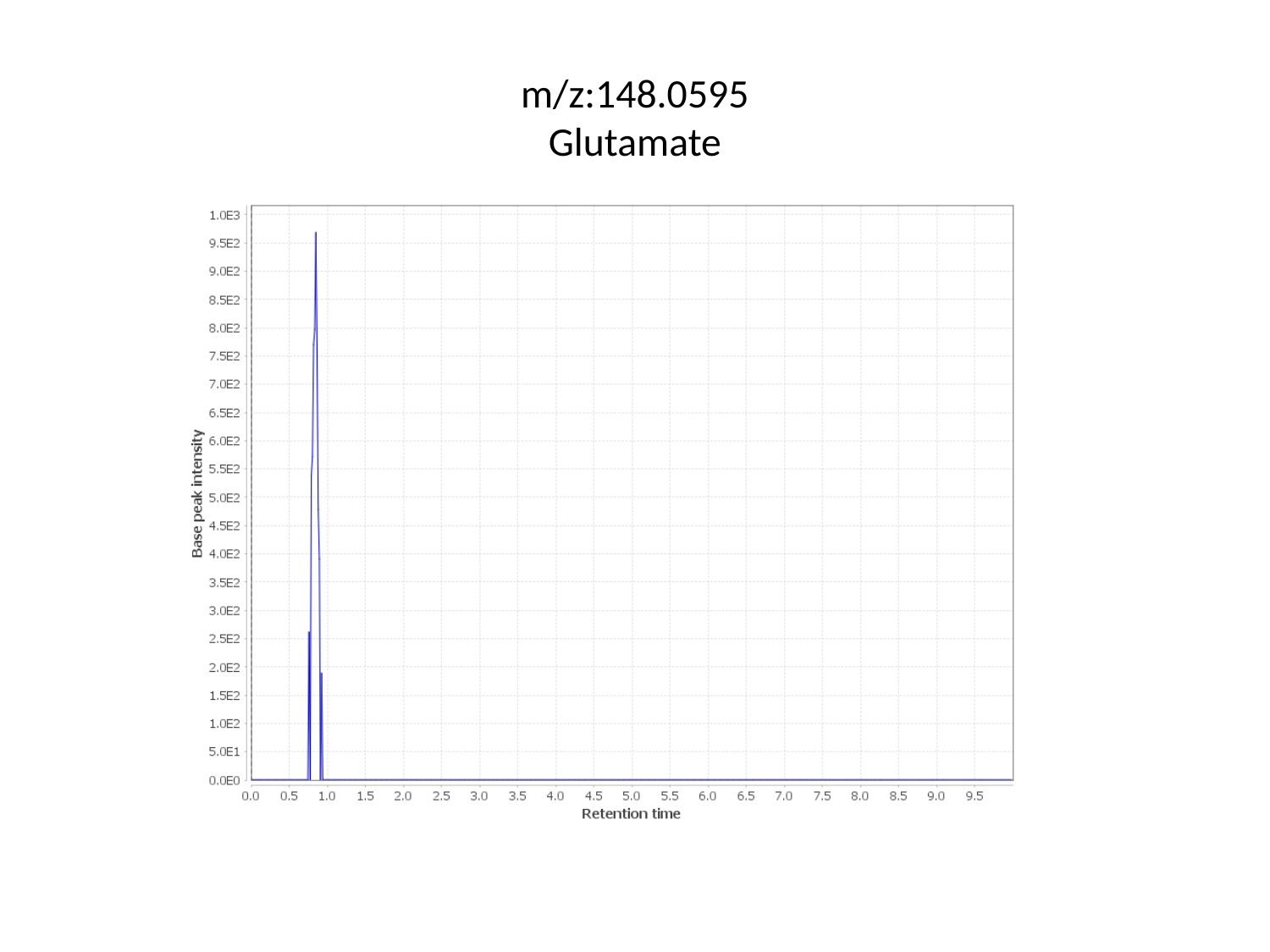

# m/z:148.0595 Glutamate

## Slide 2
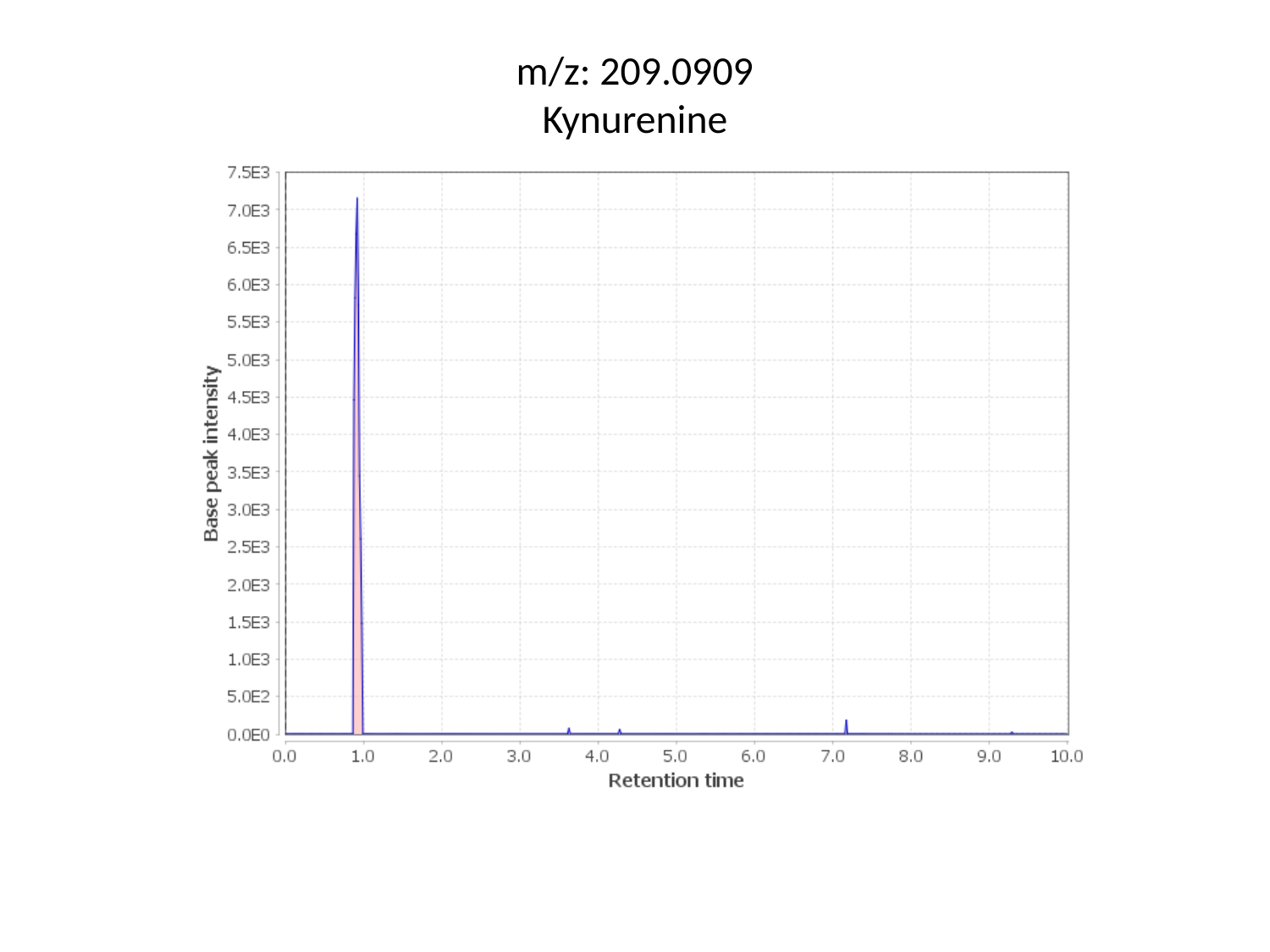

# m/z: 209.0909 Kynurenine

## Slide 3
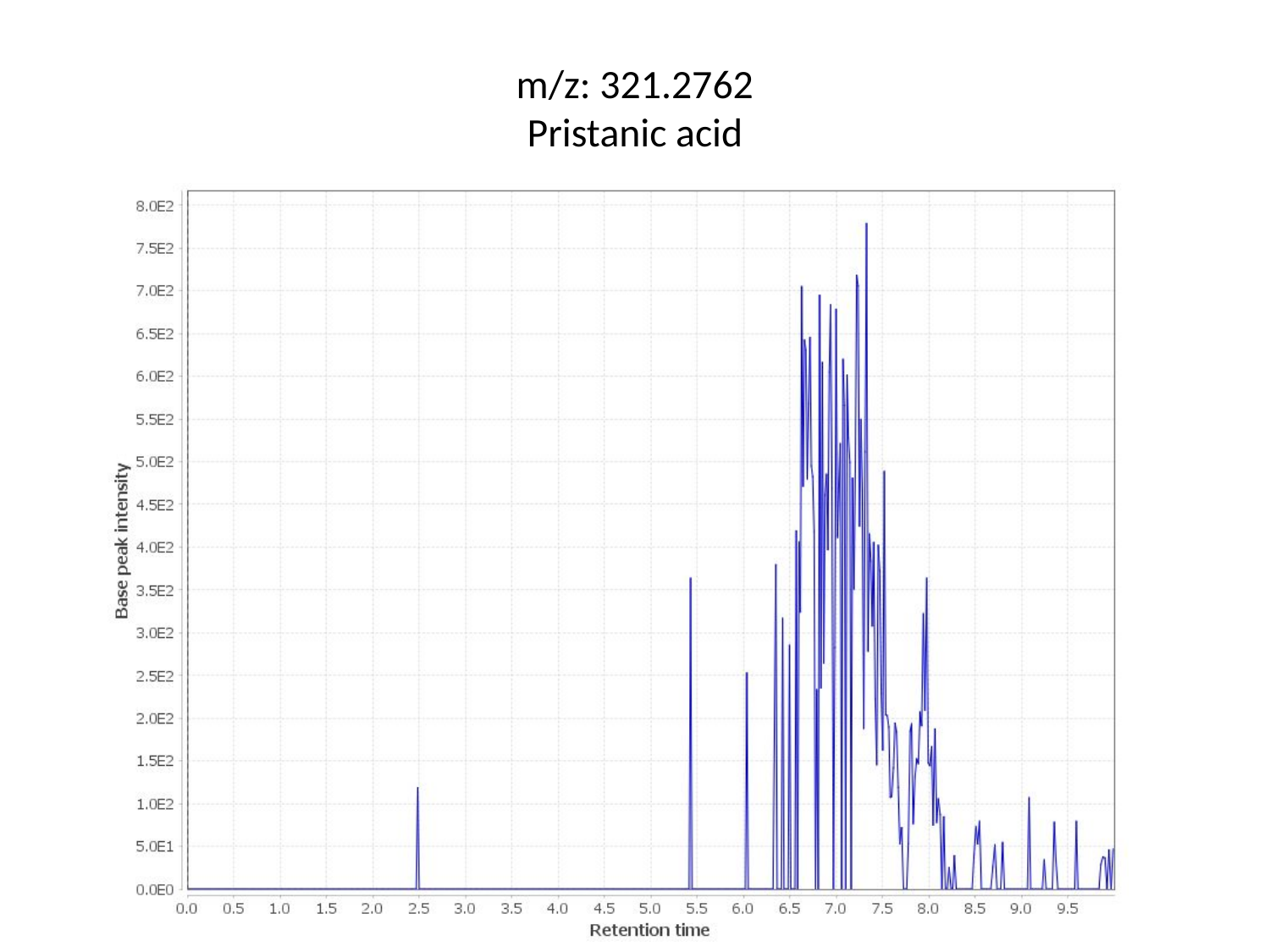

# m/z: 321.2762 Pristanic acid

## Slide 4
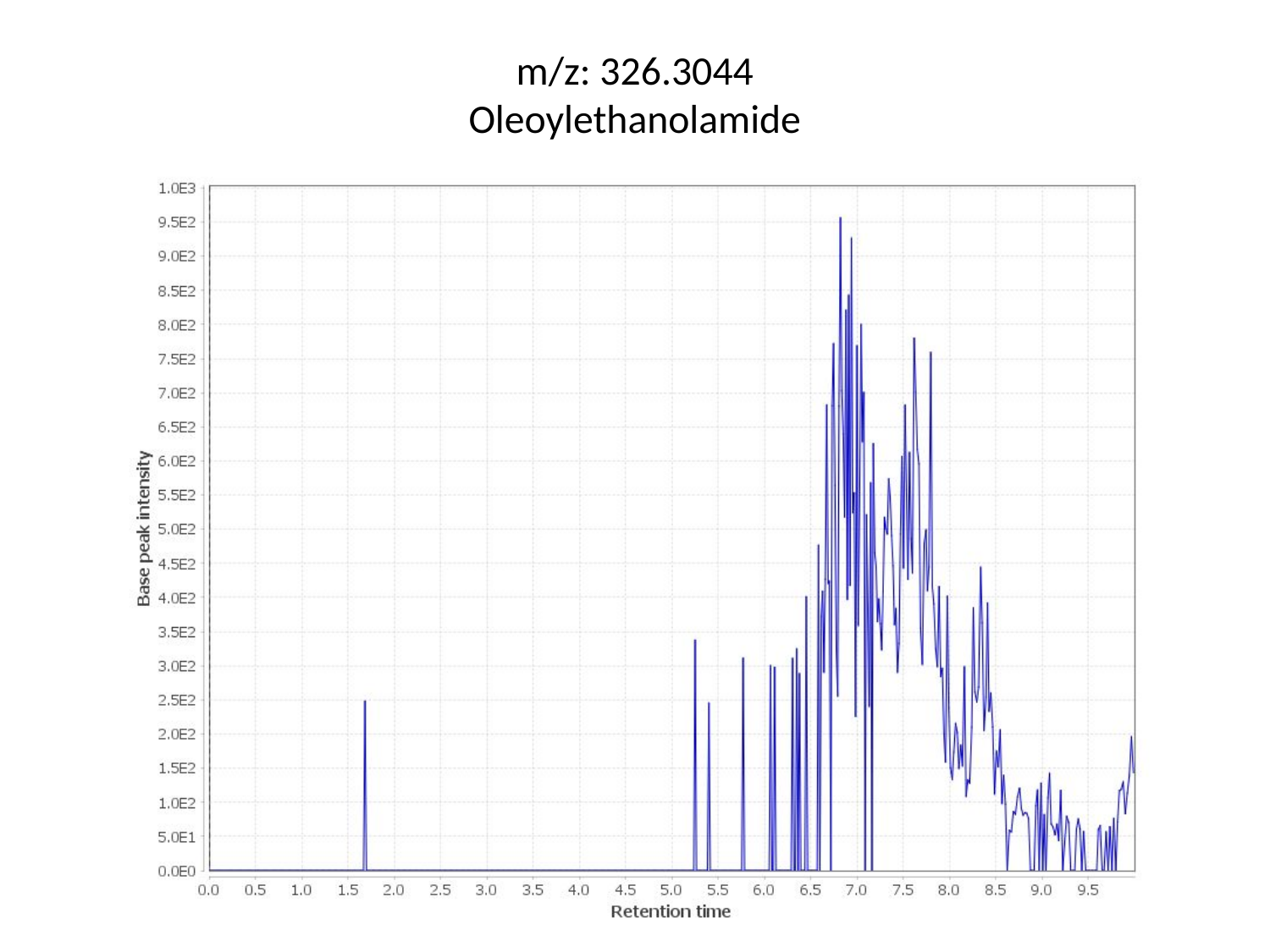

# m/z: 326.3044 Oleoylethanolamide

## Slide 5
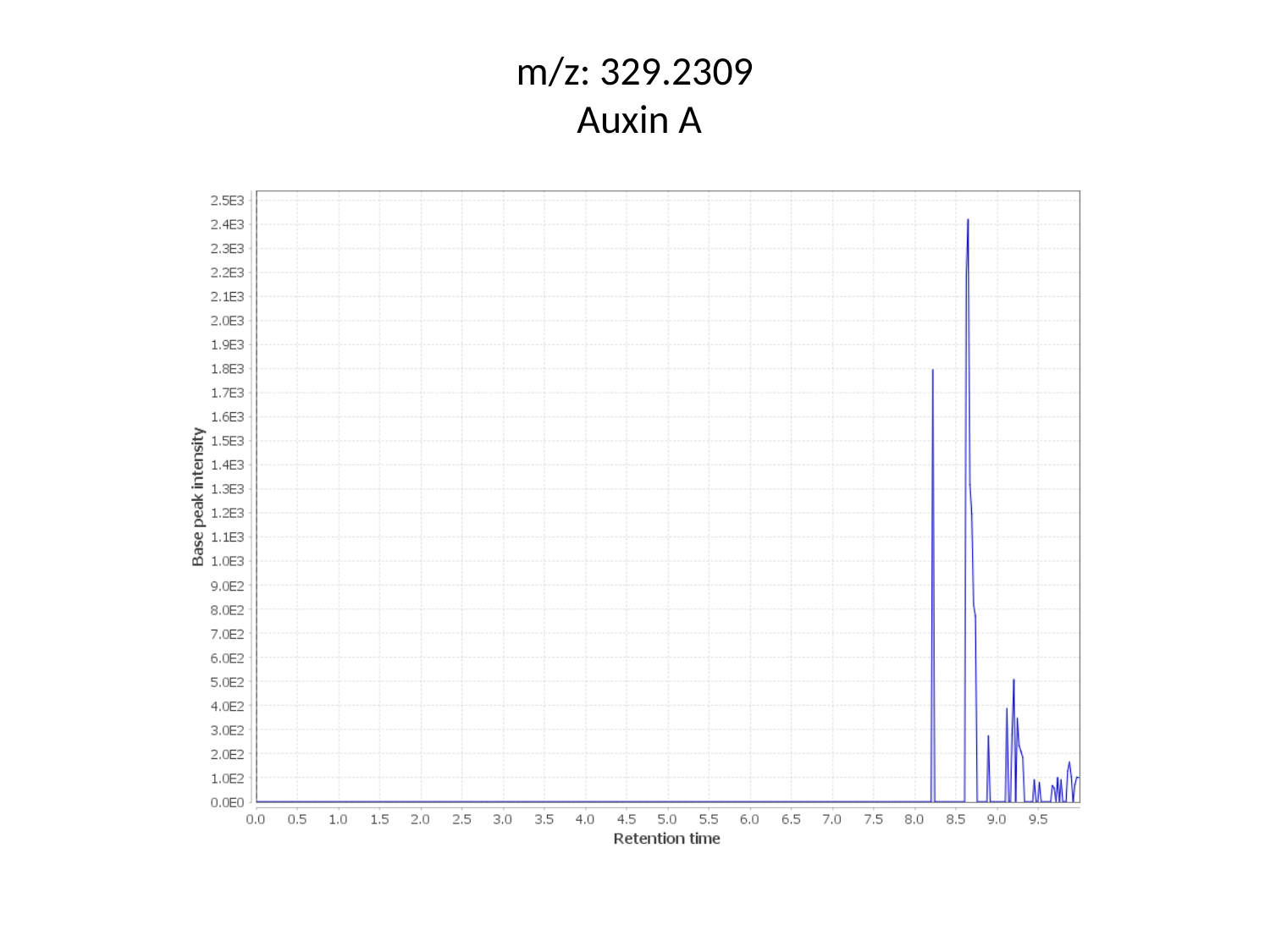

# m/z: 329.2309 Auxin A

## Slide 6
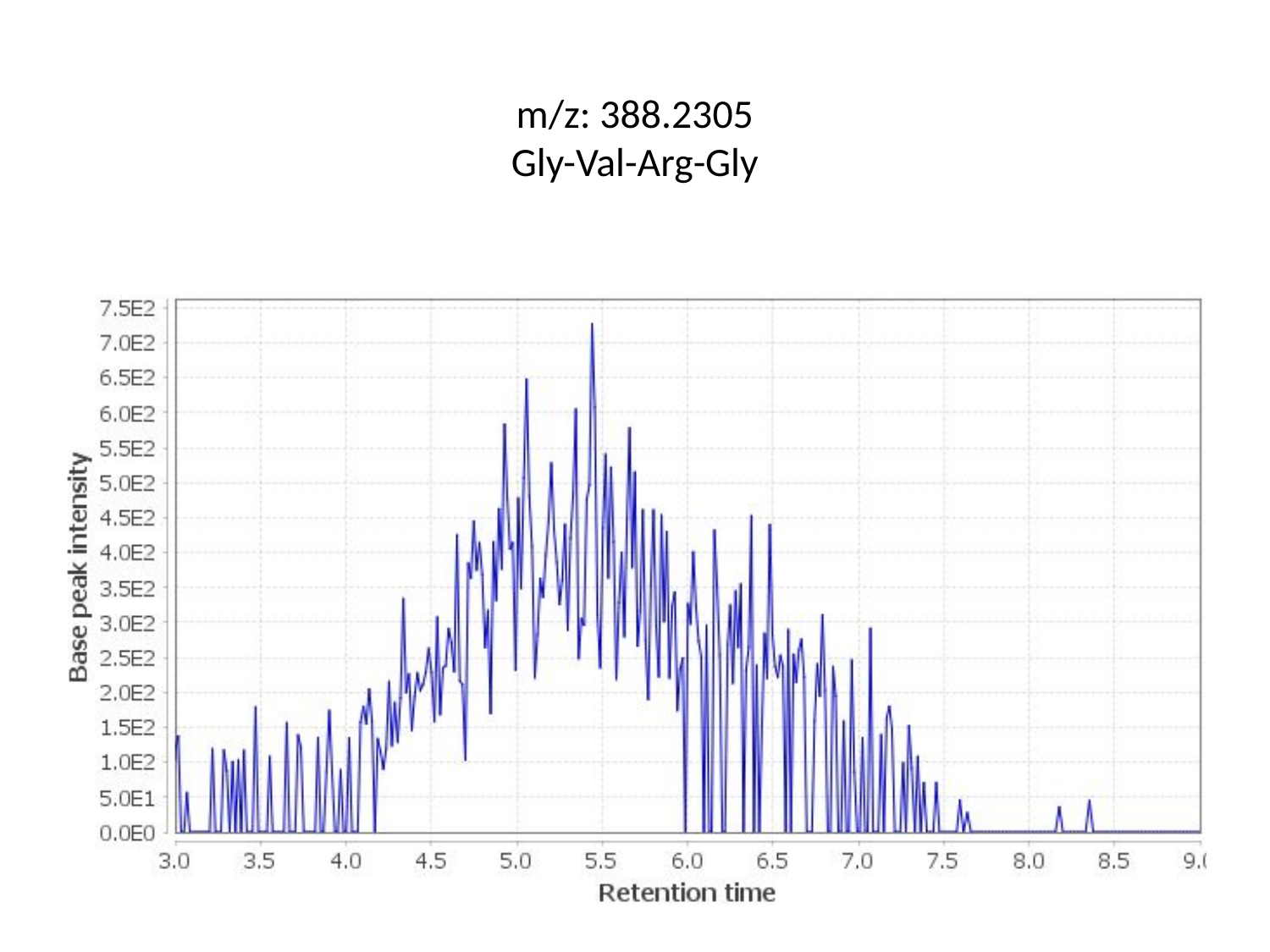

# m/z: 388.2305 Gly-Val-Arg-Gly

## Slide 7
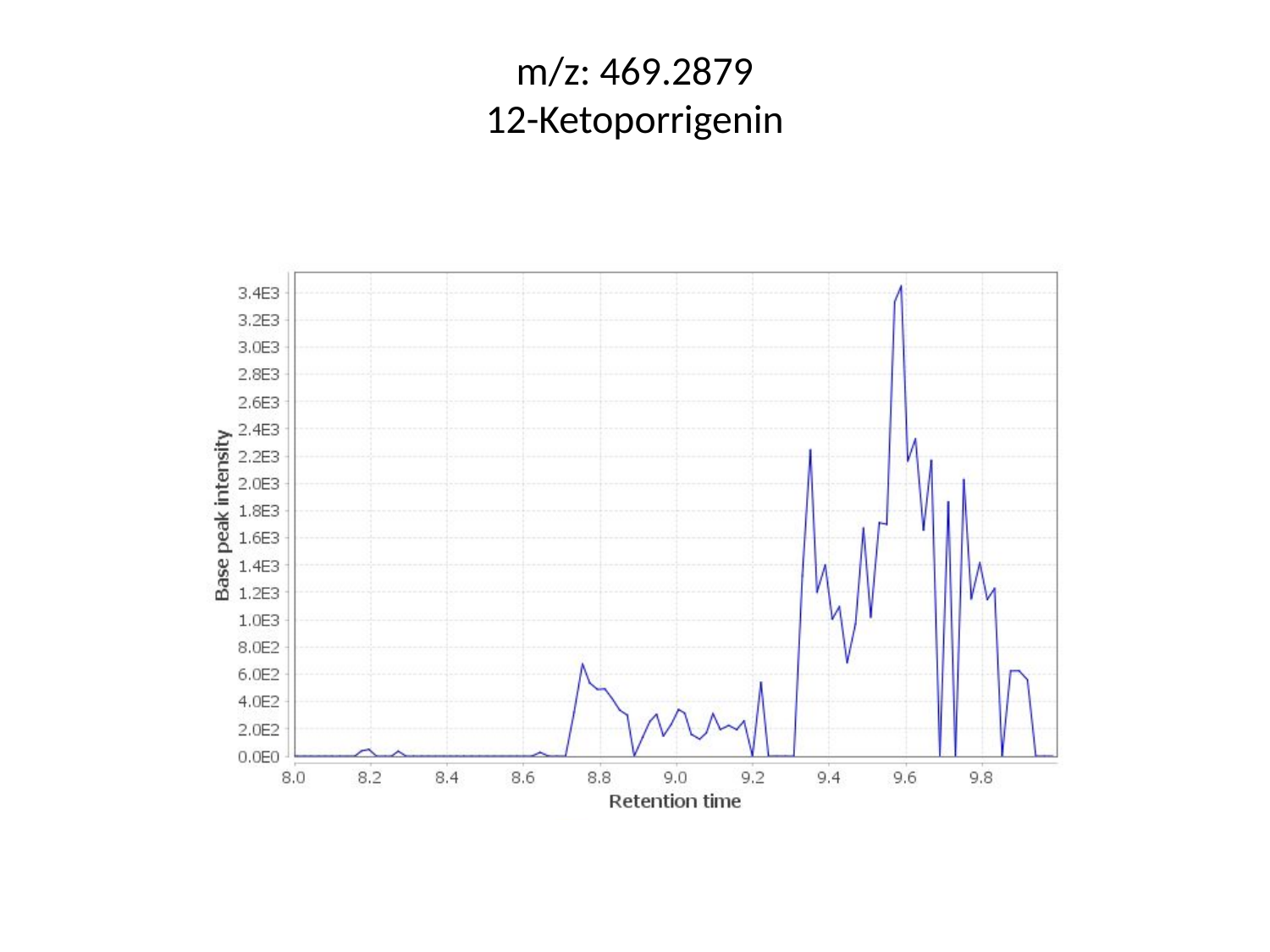

# m/z: 469.2879 12-Ketoporrigenin

## Slide 8
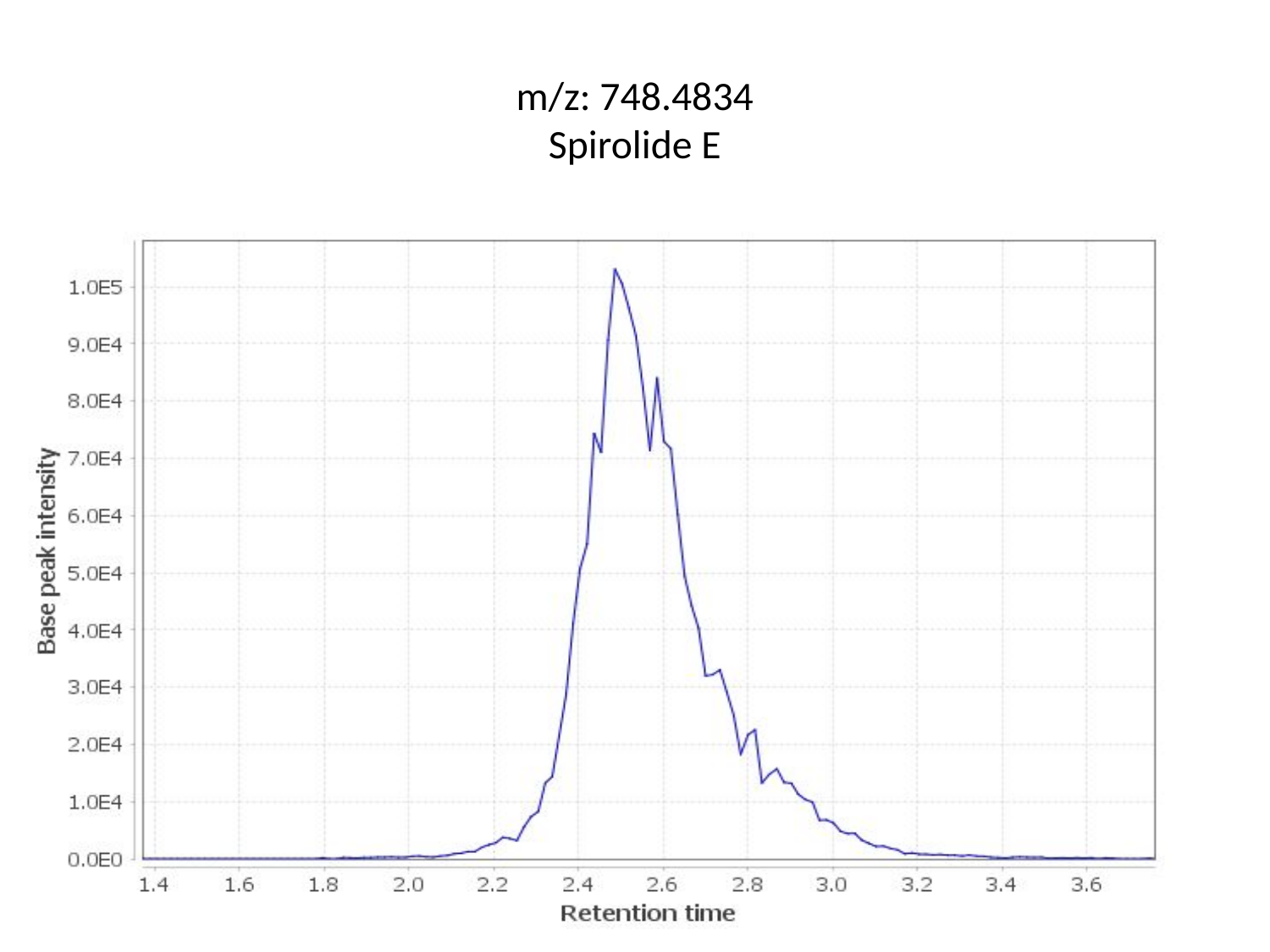

# m/z: 748.4834 Spirolide E

## Slide 9
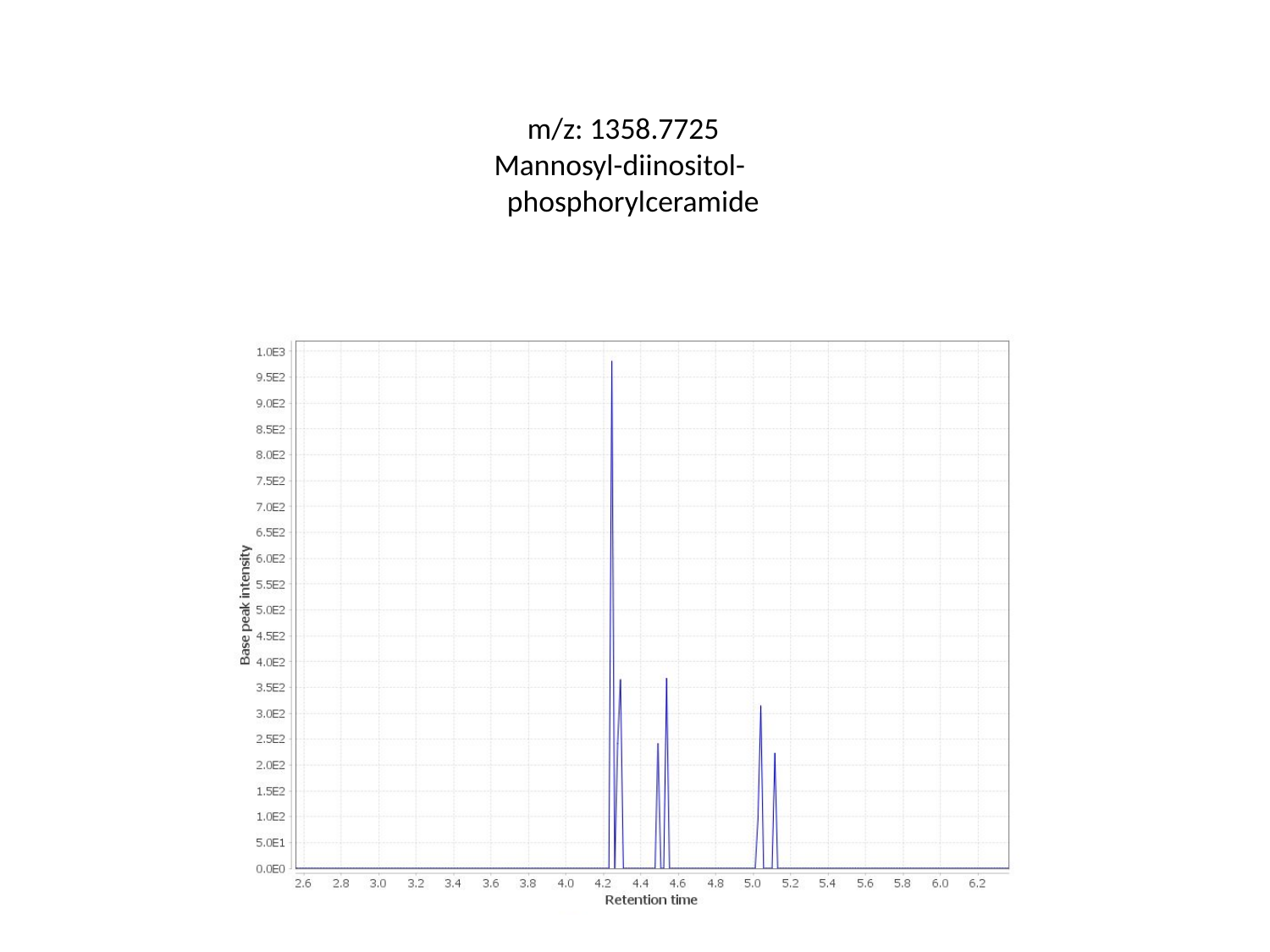

# m/z: 1358.7725 Mannosyl-diinositol- phosphorylceramide
