# Supplementary material for: Metabolic Profiles of Obesity in American Indians: The Strong Heart Family Study
Source: PLoS One. 2016 Jul 19;11(7):e0159548. doi: 10.1371/journal.pone.0159548 (PMC4951134; doi:10.1371/journal.pone.0159548)
Supplement: S1 Table — (DOCX) [file pone.0159548.s002.docx]

| S**1 Table.**  **Clinical characteristics of SHFS participants included in this study versus those who did not** | | | |
| --- | --- | --- | --- |
| Variables | Included  (n=431) | Not included  (n=1,686) | *P* value^a^ |
| Age, years | 33.9±13.4 | 35.18±15.41 | 0.12 |
| Female sex, % | 65.01 | 61.32 | 0.22 |
| Education (High school or higher), % | 62.60 | 63.00 | 0.95 |
| Body mass index, kg/m^2^ | 32.88±8.43 | 30.70±7.25 | 0.0001 |
| Waist circumference, cm | 104.15±19.14 | 99.63±15.53 | 0.03 |
| Current smoker, % | 35.93 | 36.79 | 0.75 |
| Current drinker, % | 67.14 | 64.05 | 0.37 |
| Physical activity, steps/d | 6014.13±3938.36 | 6215.22±3572.63 | 0.54 |
| Dietary protein intake, g/d | 97.51±82.98 | 99.26±79.96 | 0.26 |
| Dietary fat intake, g/d | 126.39±99.66 | 121.66±93.48 | 0.16 |
| Caloric intake, Kcal/d | 2887.59±2079.25 | 2689.36±1868.33 | 0.09 |
| Total triglyceride, mg/dL | 143.15±79.47 | 143.25±89.04 | 0.98 |
| Total cholesterol, mg/dL | 176.56±33.87 | 178.37±34.45 | 0.34 |
| HDL-cholesterol, mg/dL | 50.80±14.73 | 52.37±14.73 | 0.07 |
| LDL-cholesterol, mg/dL | 97.64±28.99 | 97.92±28.77 | 0.87 |
| Fasting glucose, mg/dL | 91.01±7.22 | 91.95±8.67 | 0.09 |
| Fasting insulin, uU/mL | 16.21±12.4 | 14.90±13.3 | 0.11 |
| HOMA-IR | 3.68±2.87 | 3.45±3.28 | 0.24 |

^a^ Family relatedness was adjusted using GEE models. HOMA-IR, homeostatic model assessment of insulin resistance; SHFS, the Strong Heart Family Study.
