# Supplementary material for: Metabolic Profiles of Obesity in American Indians: The Strong Heart Family Study
Source: PLoS One. 2016 Jul 19;11(7):e0159548. doi: 10.1371/journal.pone.0159548 (PMC4951134; doi:10.1371/journal.pone.0159548)
Supplement: S2 Table — (DOCX) [file pone.0159548.s003.docx]

**S2 Table. Metabolites significantly associated with obesity (yes/no) in American Indians**

| **Matching metabolites** | **Class** | ***m/z*** | ***Retention time* (s)** | **Model 1**^a^ | |  | **Model 2**^a^ | |
| --- | --- | --- | --- | --- | --- | --- | --- | --- |
|  |  |  |  | **Odds Ratio (95% CI)** | ***P* value** |  | **Odds Ratio (95% CI)** | ***P* value** |
| ***Positively associated metabolites*** |  |  |  |  |  |  |  |  |
| Oleoylethanolamide | Fatty amides | 326.3044 | 490 | 1.08 ( 1.02, 1.13) | 9.78×10^-3^ |  | 1.09 (1.02, 1.14) | 4.99×10^-3^ |
| Kynurenine | Amino acids | 209.0909 | 45 | 1.07 ( 1.01, 1.12) | 1.18×10^-3^ |  | 1.08 (1.02, 1.16) | 7.12×10^-3^ |
| Auxin A | Prenol lipids | 329.2319 | 30 | 1.07 ( 1.03, 1.12) | 2.64×10^-3^ |  | 1.07 (1.02, 1.13) | 8.46×10^-3^ |
| 12-Ketoporrigenin | Steroid derivatives | 469.2879 | 497 | 1.09 ( 1.03, 1.16) | 2.88×10^-3^ |  | 1.09 (1.03, 1.17) | 4.12×10^-3^ |
| *Combined effect* |  |  |  | 1.08 ( 1.06, 1.12) | 4.22×10^-4^ |  | 1.15 ( 1.09, 1.20) | 5.26×10^-5^ |
| ***Negatively associated Metabolites*** |  |  |  |  |  |  |  |  |
| Mannosyl-diinositol-  phosphorylceramide | Sphingolipids | 1358.7725 | 211 | 0.92 ( 0.87, 0.96) | 1.67×10^-3^ |  | 0.93 ( 0.88, 0.98) | 5.79×10^-3^ |

^a^ Model 1 adjusted for age, sex, site, lifestyle (smoking, alcohol drinking, and physical activity), and socioeconomic status (education level); Model 2 further adjusted for dietary intake of total calories, protein, and fat as well as insulin resistance (HOMA-IR).
