# Supplementary material for: Metabolic Profiles of Obesity in American Indians: The Strong Heart Family Study
Source: PLoS One. 2016 Jul 19;11(7):e0159548. doi: 10.1371/journal.pone.0159548 (PMC4951134; doi:10.1371/journal.pone.0159548)
Supplement: S3 Table — (DOCX) [file pone.0159548.s004.docx]

**S3 Table.** **Metabolites significantly associated with abdominal obesity (yes/no) in American Indians**

| **Matching Metabolites** | **Class** | ***m/z*** | ***Retention time* (s)** | **Model 1^a^** | |  | **Model 2^a^** | |
| --- | --- | --- | --- | --- | --- | --- | --- | --- |
|  |  |  |  | **Odds Ratio (95% CI)** | ***P* value** |  | **Odds Ratio (95% CI)** | ***P* value** |
| ***Positively associated metabolites*** |  |  |  |  |  |  |  |  |
| Glutamate | Amino acids | 148.0594 | 44 | 1.29 ( 1.06, 1.49) | 9.82×10^-3^ |  | 1.31 ( 1.05, 1.64) | 1.87×10^-2^ |
| Oleoylethanolamide | Fatty amides | 326.3043 | 490 | 1.28 ( 1.06, 1.49) | 1.10×10^-2^ |  | 1.29 ( 1.12, 1.51) | 5.41×10^-4^ |
| Kynurenine | Amino acids | 209.0909 | 45 | 1.27 ( 1.07, 1.45) | 4.96×10^-3^ |  | 1.29 ( 1.11, 1.51) | 9.31×10^-4^ |
| Gly-Val-Arg-Gly | Peptides | 388.2305 | 564 | 1.33 ( 1.15, 1.53) | 1.01×10^-3^ |  | 1.33 ( 1.08, 1.65) | 5.98×10^-3^ |
| Pristanic acid | Prenol lipids | 321.2762 | 420 | 1.12 ( 1.01, 1.21) | 2.66×10^-2^ |  | 1.07 ( 1.01, 1.14) | 1.47×10^-2^ |
| Spirolide E | Prenol lipids | 748.4834 | 426 | 1.18 ( 1.02, 1.32) | 3.62×10^-2^ |  | 1.13 ( 1.01, 1.26) | 2.69×10^-2^ |
| *Combined effect* |  |  |  | 1.23 ( 1.14, 1.33) | 2.93×10^-4^ |  | 1.21 ( 1.06, 1.38) | 6.43×10^-4^ |
| ***Negatively associated metabolites*** |  |  |  |  |  |  |  |  |
| Mannosyl-diinositol-phosphorylceramide | Sphingolipids | 1358.77 | 211 | 0.71 (0.60,0.85) | 1.69×10^-4^ |  | 0.65 (0.65,0.89) | 3.99×10^-4^ |

^a^ Model 1 adjusted for age, sex, site, lifestyle (smoking, alcohol drinking, and physical activity), and socioeconomic status (education level); Model 2 further adjusted for dietary intake of total calories, protein, and fat as well as insulin resistance (HOMA-IR).
